# The length of stay and the hospital cost for ICU patients with resistant bacterial infections: a pilot study

**DOI:** 10.1186/1471-2334-14-S3-P38

**Published:** 2014-05-27

**Authors:** Asim Priyendu, Nagappa Anantha Naik, Muralidhar Varma, Kalwaje Eshwara Vandana, Balakrishnan Rajesh

**Affiliations:** 1 Manipal College of Pharmaceutical Sciences, Manipal University, Karnataka, India; 2 Department of Medicine, Kasturba Medical College & Hospital, Manipal University, Karnataka, India; 3 Department of Microbiology, Kasturba Medical College, Manipal University, Karnataka, India; 4 School of Pharmacy. University of Michigan, MI, USA

## Background

Healthcare in India comes mostly as an out of the pocket payment as health insurance is virtually absent for majority of the population. Determination of the typical hospital cost and length of stay (LOS) are of decisive importance for patients, healthcare providers, and payers who need to make evidence based rational decisions about patient care and the allocation of resources.

## Methods

A cross sectional pilot study was performed and the data for LOS and hospital cost was collected from the medicine ICU and the accounts section for 38 patients with resistant bacterial infections who were admitted to Intensive care unit. The data was analyzed for average LOS and hospital cost for infections. The data was analyzed using univariate analysis.

## Results

Almost 29% of the patients with resistant infections were between 18-40 years of age. The mean cost of hospitalization was found to be INR76082.79 and only 34% of the patients were having any insurance coverage. The median length of stay in the hospital ICU was 8.5 days. 37% of the patients were on ventilator during the hospitalization and 25% of the total patients died.

## Conclusion

Resistant bacterial infections may lead to a high LOS in the ICU furthermore adding to the economic burden of the patient, healthcare provider and the payer. These results can be used to study the treatment alternatives in case of resistant infections and making them cost effective at the same time stressing upon the need of health insurance among the population.

